# Examining Differences in Real-World Effectiveness of e-Cigarettes for Smoking Cessation by Source of Purchase in England: An Observational Study of Different Contexts Before and After the COVID-19 Pandemic

**DOI:** 10.1093/ntr/ntae178

**Published:** 2024-07-18

**Authors:** Sharon Cox, Sarah E Jackson, Jamie Brown, Loren Kock, Lion Shahab

**Affiliations:** Department of Behavioural Science and Health, University College London, London, UK; Spectrum Research Consortium, London, UK; Department of Behavioural Science and Health, University College London, London, UK; Spectrum Research Consortium, London, UK; Department of Behavioural Science and Health, University College London, London, UK; Spectrum Research Consortium, London, UK; Vermont Center on Behavior and Health, University of Vermont, Burlington, USA; Department of Behavioural Science and Health, University College London, London, UK; Spectrum Research Consortium, London, UK

## Abstract

**Introduction:**

The effectiveness of e-cigarettes for smoking cessation may differ by source of purchase. The changing influence of self-selection on purchase location caused by COVID-19 pandemic-related vape shop closures means we can examine the association between smoking abstinence e-cigarette use by purchase source and test for the moderation of this association by the timing of the pandemic.

**Aims and Methods:**

Repeat-cross-sectional nationally representative surveys, conducted between January 2017 and August 2023. Participants (*N* = 1284; ≥18 years, 46.9% women) who made a past-year quit attempt, used an e-cigarette in their most recent attempt, currently vaping and provided data on the purchase source were included. The association between e-cigarette purchase source and continuous abstinence following the most recent quit attempt was assessed with adjusted regression, including an interaction between the timing of pandemic restrictions (March 2020 through January 2022).

**Results:**

Overall, 48.1% usually purchase their e-cigarettes from vape shops, declining from 53.6% prepandemic to 40.6% during the pandemic. There was inconclusive evidence that those purchasing from vape shops had greater odds of quitting smoking (OR_adj_ = 1.25, 95% CI = .92 to 1.76). The association between purchase source and successful quitting did not depend upon whether purchasing occurred before or during the pandemic (*F* = 0.08, *p*_interaction_ = .774; prepandemic: OR_adj_ = 1.23, 0.79–1.91; and pandemic: OR_adj_ = 1.29, 0.81–2.06).

**Conclusions:**

More data are needed to conclusively determine whether purchasing from a specialist vape shop increases smoking cessation. Given the changing influence of self-selection on purchase location caused by the pandemic, the similar associations between purchase source and quit success across both periods suggest it is unlikely an artifact of unmeasured confounding.

**Implications:**

If purchasing e-cigarettes from a specialist vape shop can increase their effectiveness for smoking cessation is an empirical question. While we found a positive association between purchasing from a specialist vape shop and abstinence rates, the results were inconclusive. Further studies are required to establish whether purchasing from specialist vape shops improves smoking cessation outcomes compared with other purchase sources.

## Introduction

Pharmacotherapy along with behavioral support is regarded as the “gold standard” treatment for people who smoke,^1^ but it is rarely used (<1% of all quit attempts in England).^2^ While e-cigarettes have been found to be as effective as the best pharmacotherapy,^3^ they have the additional advantage of being popular, being used in ~40% of quit attempts in England.^2^ The landscape of how people choose to quit is changing and more people are opting to quit smoking without traditional pharmacotherapies and or the support of a medical or stop-smoking practitioner, opting instead to purchase e-cigarettes from convenience stores or from specialist e-cigarette retailers (vape shops hereafter).^4^ Vape shops are a common source of purchase of e-cigarettes, used by ~40% of vapers.^5^ Vape shops differ from other e-cigarette retailers (including supermarkets, convenience stores, petrol stations, and some online) in offering specialist advice on product choice^6^ and ongoing support and troubleshooting, which may reduce the risk of relapse due to user error or device malfunction.^7^ Multiple experimental^8–10^ and observational studies^11,12^ have found e-cigarettes to be effective in increasing quit rates. Whether their effectiveness differs according to the source of purchase is not known. If purchasing from a particular source (eg, a vape shop) is more effective in helping people quit and stay quit, then this is an important public health message for smokers looking to quit smoking with an e-cigarette. This study examines the real-world effectiveness of e-cigarettes purchased from specialist vape shops compared with those purchased from other sources, using the COVID-19 pandemic as a natural experiment to explore the possible impact of selection bias.

Vape shops are commonplace in England. Unlike other e-cigarette retailers, vape shops offer a large range of products including specialist products (eg, self-build mod devices) as well as e-liquids that are not commonly found within convenience stores. There are several reasons why vape shops may help people to quit more successfully than other less specialized retailers. First, many offer an “expert by experience” service, staff are often e-cigarette users and ex-smokers who use their personal experiences to guide customers.^6,7,13,14^ Second, in surveys of British adults who have tried vaping, low satisfaction is a common reason for discontinuing use,^15^ and it is plausible that vape shop staff who have successfully quit may be able to deal with these concerns by advising on nicotine strength, device power, and flavors. Third, qualitative research has highlighted that having advice from a specialist may help inexperienced users navigate e-cigarette purchasing.^7^ However, given nicotine is the primary reinforcer for satisfaction,^16^ finding a device with good pharmacokinetics may supersede the extra-services that vape shops offer in helping people quit smoking.

While growing evidence suggests vape shops can play a role in helping people to stop smoking, it is not necessarily true that all vape shops offer a tailored experience or one that is necessarily focused on helping the consumer to fully quit smoking. Vape shops that take part in scientific research may not be representative of the wider category, which may cause qualitative studies to overestimate their positive effects. Population-based data collected from consumers can provide useful triangulation.

Since November 2006, the representative Smoking Toolkit Study has been monitoring smoking cessation activity in the general population in England each month.^17^ We have an established method of assessing real-world effectiveness of cessation aids,^11,12,18–20^ which involves comparing the success rates of smokers trying to quit with different methods and adjusting statistically for a wide range of factors that could potentially bias the results.^21^ We have used this method to assess the overall real-world effectiveness of e-cigarettes,^11^ and there are now data in the Smoking Toolkit Study to enable comparative estimates of effectiveness by source of purchase.

During the COVID-19 pandemic, England went into “lockdown” and only essential stores were allowed to remain open.^22^ Vape shops were used less frequently during the pandemic^4^: Some were forced to close for brief periods as nonessential shops—although a small proportion of vape shops did remain open in some form, offering telephone advice and sending out products^23^; a number went out of business; and people generally reduced visits to in-person shops. At the same time, people could still easily purchase e-cigarettes from supermarkets, convenience stores, petrol stations, or online retailers. This effectively forced a proportion of those who would otherwise have likely purchased their e-cigarette from a specialist vape shop to purchase from other sources. Therefore, the COVID-19 pandemic provides an opportunity to compare the real-world effectiveness of e-cigarettes by source of purchase while reducing self-selection bias.

A key issue with comparing the effectiveness of e-cigarettes by the source of purchase is the potential for self-selection bias,^24^ whereby smokers who purchase their e-cigarettes from different sources differ on characteristics that are related to the likelihood of successful quitting, some of which are measurable and known (eg, level of dependence, social grade, and age) but others maybe not and unknown (unmeasured confounding). In this study, we will compare estimates of effectiveness by source of purchase before the pandemic (when vape shops were open) to those during the pandemic (when access to specialist “bricks and mortar” vape shops was reduced): If the pattern of results are similar despite this changing pressure on self-selection, then the implication is that any estimates of association between source of purchase and effectiveness are more likely to reflect a genuine impact of the source of purchase (and are less likely an artefact of unmeasured confounding).

It is also important to understand whether any differences in the effectiveness of e-cigarettes by the source of purchase are caused by differences in the types of devices people buy from different outlets. Vape shops offer a wider range of products than supermarkets and convenience stores and provide customers with tailored advice on what nicotine concentration to use (4). We will compare estimates of effectiveness by source of purchase across people using different device types and nicotine concentrations to explore the extent to which these factors explain any differences in effectiveness by source of purchase.

To summarize, this study examined the association between abstinence and use of e-cigarettes by source of purchase, and whether this depends on the timing of the COVID-19 pandemic. Specifically, we addressed the following research questions:

1. Using data from England between 2017 and 2023, to what extent does the real-world effectiveness of e-cigarettes differ by source of purchase (specialist vape shop vs. other retailers)?
2. Are any differences in effectiveness of e-cigarettes by source of purchase consistently observed before the COVID-19 pandemic (when vape shops were open; data from January 2017 to February 2020) and during the pandemic (when purchasing from vape shops was reduced; data from March 2020 to January 2022)?
3. In sensitivity analyses, if we observe any differences in the effectiveness of e-cigarettes by source of purchase, are differences consistent across (a) different types of e-cigarettes and (b) nicotine concentrations (between 2017 and 2023)?

## Materials and Methods

### Design

The Smoking Toolkit Study is an ongoing monthly survey designed to provide information about smoking prevalence and factors associated with cessation in England at a population level.^17^ The study uses hybrid random location and quota sampling to select a new sample of approximately 1700 adults aged ≥ 18 years each month. For the study period before the pandemic, participants completed a face‐to‐face computer‐assisted survey with a trained interviewer. From April 2020 telephone interviews replaced face-to-face interviews but the majority of the questions and the sampling method remained the same and were similar across methods.^25^ Full details of the study’s methods are available elsewhere, and comparisons with national data indicate that key variables such as socio-demographics and smoking prevalence are nationally representative.^17^ We preregistered the protocol on the Open Science Framework (OSF) (https://osf.io/qsc4f/).

### Sample

We used data from respondents to the survey in the period January 2017–August 2023, who smoked cigarettes (including hand-rolled) or any other tobacco product (eg, pipe or cigar) daily or occasionally at the time of the survey or during the preceding 12 months. We included those who reported (i) having made at least one quit attempt in the preceding 12 months, assessed with the question “*How many serious attempts to stop smoking have you made in the past 12 months? By serious I mean you decided that you would try to make sure you never smoked again*,” (ii) using an e-cigarette in their most recent quit attempt, and (iii) being a current vaper (because only current vapers were asked the source of purchase question). Asking about e-cigarette use in the most recent attempt is an established approach to assess real-world effectiveness in England using this dataset, which has replicated many effect sizes identified in RCTs for many treatments (eg,^12,26^). Since April 2022, the item assessing the source of purchase has not been included in 10 waves due to restricted research funding; we, therefore, excluded participants surveyed in these waves.

Ethical approval for the STS has been granted by the UCL Ethics Committee (ID 0498/001). The data are not collected by UCL and are anonymized when received by UCL.

## Measures

### Outcome Variable: Self-Reported Abstinence

The outcome of interest was self-reported continuous abstinence from the start of the most recent quit attempt up to the time of the survey. Respondents were asked, “*How long did your most recent quit attempt last before you went back to smoking?*” Responses were coded 0 for those reporting a duration of time before they relapsed and 1 for those who responded that they were still not smoking.

### Explanatory Variable: Use and Source of e-Cigarette Purchase

The explanatory variable was the source of e-cigarette purchases. Current vapers were asked to choose one from the question, “*From where do you usually buy your e-cigarettes*?” Options are (i) specialist vape\electronic cigarette retailer, (ii) online vape retailer, (iii) other online retailers, (iv) newsagent\off licence\corner shop (ie, convenience store), (v) petrol garage shop, (vi) supermarket, (vii) buy them cheap from friends, (viii) other, and (ix) don’t know. As we were only interested in specialist bricks-and-mortar vape stores, we excluded online vape retailers from our primary analysis; however, in an unplanned sensitivity analysis, these were included along with the specialist vape shops. Responses were coded 1 for the vape shop (option (i) above) and 0 for all others.

### Confounding Variables

Level of cigarette addiction was assessed by self-reported ratings of the strength of urges to smoke over the last 24 h (*not at all* (coded 0), *slight* (1), *moderate* (2), *strong* (3), *very strong* (4), *extremely strong* (5)). This question was coded “0” for smokers who responded “not at all” to the question “*How much of the time have you spent with the urge to smoke?*.”

We also included variables relating to the most recent quit attempt, including time since the attempt was initiated (<1, 1–6, and >6 months), the number of quit attempts prior to this attempt that occurred in the last 12 months, use of an e-cigarette in any prior quit attempt, whether the quit attempt was planned, whether the respondent cut down first or stopped abruptly and use of other evidence-based smoking cessation aids in the most recent quit attempt (ie, prescription or over-the-counter NRT, behavioral support, or the medications varenicline, bupropion and telephone/written, eg, self-help materials/digital, eg, website and apps), coded no (0), yes (1).

The socio-demographic variables assessed were age (continuous), sex (women vs. other (men and in another way combined)) and social grade (ABC1, which includes managerial, professional and intermediate occupations, vs. C2DE, which includes small employers and own‐account workers, lower supervisory and technical occupations, and semi‐routine and routine occupations, never workers, and long‐term unemployed).

### Moderator Variable: Pandemic Timeframe

To assess the impact of the pandemic on the effectiveness outcomes for research question 2, we included a variable coded 0 before and 1 during the pandemic as a moderator. The prepandemic period specified for this analysis was January 2017 through February 2020 and the time during the pandemic was March 2020 through January 2022 (based on infection rates and restrictions, January 2022 was considered the latter end of the impacts caused by the pandemic in the United Kingdom).

### Moderator Variables: Device Type and Nicotine Strength

For research question 3, we assessed device type and nicotine strength. Device type was assessed with the question: “Which of the following do you mainly use…?” Response options were: (i) Disposable—“A disposable e-cigarette or vaping device (nonrechargeable),” (ii) Refillable—“An e-cigarette or vaping device with a tank that you refill with liquids (rechargeable)” or “A modular system that you refill with liquids (you use your own combination of separate devices: batteries, atomizers, etc.),” (iii) Pod—“An e-cigarette or vaping device that uses replaceable prefilled cartridges (rechargeable).” These were categorized as (i) refillable (reference category), (ii) disposable, and (iii) pod.

Nicotine concentration was assessed with the question: “Does the electronic cigarette or vaping device you mainly use contain nicotine?” with response options yes, no, and don’t know. Those who responded yes to this question were then asked: “What strength is the e-liquid that you mainly use in your electronic cigarette or vaping device?” (i) No nicotine, 6 mg/mL (~0.6%) or less, (ii) 7 mg/mL (~0.7%) to 11 mg/mL (~1.1%), (iii) 12 mg/mL (~1.2%)–19 mg/mL (~1.9%), (iv) 20 mg/mL (~2.0%) or more, and (v) Don’t know. For analysis, we collapsed these to: (i) No nicotine, (ii) 1–6 mg/mL nicotine, (iii) 7–12 mg/mL and, (iv) 13+ mg/mL and don’t know were excluded (<0.3%). We had not planned in our protocol to treat any nicotine as its own category, but after discussion, we decided because we are looking at the association with not smoking that it was important to distinguish this as its own category.

### Analyses

Weighted descriptive statistics (% (*n*) or means ± standard deviations (SDs), 95% confidence intervals (CI)) as appropriate) are reported for the variables included in the analyses.

For research question 1, we conducted unadjusted and adjusted (for cessation aids and the covariates specified above) logistic regressions to test the association between e-cigarette source of purchase and abstinence, aggregated across all waves of data available for this study.

For research question 2, we conducted unadjusted and adjusted logistic regressions to test the two-way interaction between the source of e-cigarette purchase (specialist vape retailer vs. all others) and the timing of the pandemic (prepandemic vs. pandemic period) on abstinence (abstinent yes vs. no). In an unplanned sensitivity analysis, we also included online vape retailers in the specialist vape shops category and reran this analysis. This was to explore whether across the broadest category of specialist vape retailers, there were any associations with abstinence.

A planned sensitivity analysis, including device type and nicotine strength, is included for research question 3. We conducted unadjusted and adjusted logistic regressions to test the two-way interaction between the source of e-cigarette purchase (specialist vape retailer vs. all others) and the device type in one model, and nicotine strength in another, on abstinence (abstinent yes vs. no) to explore whether any association between source of purchase and abstinence differed across device types or nicotine strengths.

To aid in the interpretation of nonsignificant results, we calculated Bayes factors (half-normal distribution; prior estimated effect size OR 1.4 as per Cochrane review for behavioral support only,^27^) for nonsignificant results pertaining to the main effects of source of purchase.

All analyses were performed using R Studio (R version 4.2.2).

## Results

A total of 130 111 (unweighted) adults aged ≥ 18 years in England were surveyed between January 2017 and August 2023, of whom 7495 had attempted to quit smoking in the past year. Of this group, 2370 (31.6%) reported using an e-cigarette in their most recent quit attempt and 1849 (78.0%) of them were still vaping at the time of the survey. We excluded 305 participants surveyed in waves in which the source of purchase of e-cigarettes was not assessed and a further 53 with missing data on the source of purchase. For our primary analyses, we also excluded 207 who reported buying from online vape retailers, leaving a final sample of 1284 participants (weighted *n* = 1364). Approximately half (48.1%) reported that they usually bought their e-cigarettes from vape shops, with the percentage declining from 53.6% prepandemic to 40.6% during the pandemic, while the remainder bought from other sources. The only statistically significant differences between the groups were that of device type and nicotine strength, this is likely driven by disposables as they are more commonly purchased in other (nonvape shop) locations and generally with higher nicotine strength. Sample characteristics are shown in Table 1.

**Table 1.** Weighted Sample Characteristics by Usual Source of Purchase

	Vape shop (*n* = 667)	Other (*n* = 697)	*p*
**Sociodemographic characteristics**			
Age, mean (SD)	37.5 (14.0)	39.1 (14.9)	.124
Women	311 (46.6%)	328 (47.1%)	.862
Social grade C2DE	399 (59.8%)	421 (60.5%)	.806
**Vaping characteristics**			
Device currently using			**<.001**
Disposable	34 (5.1%)	133 (19.4%)	
Refillable	559 (84.6%)	398 (57.9%)	
Pod	68 (10.4%)	156 (22.7%)	
Nicotine strength currently using			**<.001**
0	82 (12.5%)	90 (14.0%)	
1–6 mg/mL	318 (48.7%)	236 (36.4%)	
7–11 mg/mL	69 (10.6%)	78 (12.1%)	
≥12 mg/mL	184 (28.2%)	243 (37.5%)	
**Smoking characteristics**			
Strength of urges to smoke, mean (SD)	1.8 (1.1)	1.7 (1.2)	.340
**Quit attempt characteristics**			
Time since the most recent attempt started			.687
<1 month	117 (17.7%)	132 (18.9%)	
1–6 months	327 (49.2%)	324 (46.6%)	
>6 months	220 (33.1%)	240 (34.4%)	
The most recent attempt was unplanned	354 (53.6%)	383 (56.0%)	.431
The most recent attempt was abrupt (did not cut down first)	338 (50.9%)	366 (52.7%)	.543
The most recent attempt involved the use of other aids	88 (13.2%)	112 (16.0%)	.174
Number of past-year quit attempts			.467
1	424 (63.6%)	444 (63.8%)	
2	147 (22.0%)	135 (19.4%)	
3	43 (6.4%)	60 (8.6%)	
4	53 (8.0%)	57 (8.3%)	
Use of an e-cigarette in any prior quit attempt	196 (29.4%)	196 (28.1%)	.646

Abbreviation: SD = standard deviation.

Data are presented as weighted *n* (%), unless otherwise specified. There were some missing data on some variables (gender *n* = 1, device type *n* = 16, nicotine strength *n* = 63, strength of urges *n* = 12, time since most recent quit attempt started *n* = 4, most recent quit attempt was unplanned *n* = 18, most recent quit attempt was abrupt *n* = 4); sample sizes do not sum to the total on these variables, valid percentages are shown. *p*-Values highlighted in bold indicate a statistical significance value of <.001. The quit success rate was 25.6% (95% CI = 22.2% to 29.0%) among those who reported purchasing their e-cigarettes from specialist vape shops and 23.4% (95% CI = 20.2% to 26.6%) among those purchasing from other sources. Table 2 summarizes the results of the logistic regression models. Overall, those reporting vape shop purchases had 25% higher odds of successful quitting after adjustment for covariates, but this was not significant (OR_adj_ = 1.25, 95% CI = .92 to 1.76, *p* = .156). The interaction between the source of purchase and the timing of the pandemic was not significant (*F* = 0.08, *p* = .774), and the association was similar among those surveyed prepandemic (OR_adj_ = 1.23, 95% CI = .79 to 1.91, *p* = .368) and during the pandemic (OR_adj_ = 1.29, 95% CI = .81 to 2.06, *p* = .278). The calculation of a Bayes factor for this association, once fully adjusted, indicated that the data were insensitive to distinguish no effect from the hypothesized true effect size (OR = 1.40) if it existed (Table 2). As there were no significant main effects, we did not conduct the analyses for device type nor nicotine strength, as these were conditional on these results.

**Table 2.** Associations Between the Source of Purchase and Effectiveness of e-Cigarettes for Smoking Cessation

	Unadjusted			Adjusted^a^		
	OR [95% CI]	*p*	BF^b^	OR [95% CI]	*p*	BF^b^
**Vape shop [ref other]**						
Overall^c^	1.16 [0.88–1.53]	0.292	1.03	1.25 [0.92–1.72]	0.156	1.66
Prepandemic^d^	1.43 [0.98–2.10]	0.063	3.28	1.23 [0.79–1.91]	0.368	1.16
Pandemic^e^	0.99 [0.65, 1.51]	0.972	0.52	1.29 [0.81–2.06]	0.278	1.36

Abbreviations: BF = Bayes factor; CI = confidence interval; OR = odds ratio.

^a^Adjusted for age, sex, social grade, time since most recent quit attempt started, whether most recent quit attempt was planned, stopped abruptly in most recent quit attempt, use of evidence-based aids in most recent attempt, number of past-year quit attempts, and use of an e-cigarette in any prior attempt.

^b^Bayes factor based on an expected effect size of OR = 1.4. Values below 1/3 indicate the data provides evidence for the null hypothesis, values above 3 evidence for the alternative hypothesis, and values between 1/3 and 3 indicate the data are insensitive.

^c^January 2017 through August 2023.

^d^January 2017 through February 2020.

^e^March 2020 through January 2022.

An unplanned sensitivity analysis in which we expanded the definition of vape shops to include online vape retailers showed a similar pattern of results (main effect of source of purchase: OR_adj_ = 1.23, 95% CI = .92 to 1.65, *p* = .168, BF = 1.62; interaction between source of purchase and the timing of the pandemic: *F* = 0.67, *p* = .412).

## Discussion

Among people who reported stopping smoking with an e-cigarette in England, there is inconclusive evidence that those who reported usually purchasing their products at specialist vape stores had higher odds of being abstinent after adjustment for a range of socio-demographic, smoking, and quitting characteristics. The pattern of results was the same when online vape retailers were included within the category of specialist vape shops. Insofar that people who purchase from vape shops are more likely to report being abstinent, it is possible that the result may arise from unmeasured confounding (ie, people who choose to purchase from vape shops are more likely to quit successfully in ways that were unable to adjust for). However, the COVID-19 pandemic provided an opportunity to evaluate this possibility: Vape shops were used less frequently during the pandemic due to various restrictions and changes in behavior—and we observed a decline from 53.6% to 40.6%—which effectively forced a proportion of those who would otherwise have likely purchased their e-cigarette from a specialist vape shop to purchase from other sources. Despite this enforced change in the types of people purchasing their devices from vape shops, the association between source of purchase and quit success was similar before and during the pandemic period, which suggests it is less likely to be an artefact of unmeasured confounding.

Our evidence adds to the small number of studies that have explored the role that vape shops play in helping people to quit smoking. A number of qualitative studies have suggested they can have a beneficial impact on helping people to tailor the best products for their needs when they decide to quit.^6,7^ One issue for these studies is that those who are seeking tailored advice from a vape shop may be more motivated than those who buy their products elsewhere, and the sample sizes are often small. One recent larger online randomized optimization experiment showed that offering tailored advice on flavors along with text message support increased the odds of 12-week abstinence, but advice on device, nicotine strength, and relative harms did not.^28^ However, this study involved an online vape retailer. We may expect some advice, and face-to-face advice to be better than none. Our results indicate there may be *some* benefits for *some* people from buying from a specialist vape shop, what we do not show is which subgroups, ie, which demography most benefits and who does not. Moving forward, larger samples, as well as randomized controlled studies, are needed to provide a clearer indication as to the nature of these associations.

Strengths of the study include data being drawn from a national household survey and repeated data being collected throughout the pandemic period. There were also some limitations. We only included those who were currently using an e-cigarette (because only these people were asked about their source of purchase), so those who tried to quit with an e-cigarette and then stopped vaping before the survey were not included. This could introduce selection bias, however, research on the use of e-cigarettes for a quit attempt suggests that a large proportion use these for over one year suggesting that only a small proportion of people would be affected by this selection criteria.^9^ Another limitation is the potential imprecision of the word “usually” regarding the source of purchase; respondents may still have indicated specialist vape shops even though these were not commonly open during the pandemic. The questions about the source of purchase, main device type and nicotine concentration refer to current behavior, which may be different to behavior when the quit attempt occurred. Lastly, our questions rely on self-reporting which can introduce recall and desirability bias.

Future research could attempt to remediate some of these issues, as well as revisit this analysis when more data have been collected. Furthermore, future research should investigate the effectiveness of e-cigarettes for smoking cessation by source of purchase including when offered with behavioral support from a trained health practitioner or similar. Such research could also assess, as a secondary outcome, the extent to which long-term vaping varied by these settings, as vape shops have a commercial interest in promoting the use of e-cigarettes beyond the period for which they may be necessary for smoking cessation. Hajek et al., have shown that e-cigarettes were twice as effective for quitting for up to one year when offered with behavioral support compared with nicotine replacement by the stop-smoking service. Many people will still benefit from face-to-face advice within a medicalized setting, and there is a role for stop-smoking services in offering e-cigarettes and other products to those who feel they do not want to use or cannot use vape shops.^7^ Stop-smoking services may also be more able to offer advice for those who want to stop or reduce their vaping. However, any potential benefits specialist vape shops may offer, needs to be considered alongside potential unintended consequences, such as convenience sales to nonsmokers.

In conclusion, from data collected between 2017 and 2023 in England, we found a positive association between purchasing from a specialist vape shop and abstinence rates, the results were inconclusive. Further studies are required to establish whether purchasing from specialist vape shops improves smoking cessation outcomes compared with other purchase sources.

## Data Availability

The selected data used for this study is available on request to the corresponding author (SC). The code used for this study will be made available via the OSF following publication.
